# Evaluation of the precision of contrast sensitivity function assessment on a tablet device

**DOI:** 10.1038/srep46706

**Published:** 2017-04-21

**Authors:** Michael Dorr, Luis A. Lesmes, Tobias Elze, Hui Wang, Zhong-Lin Lu, Peter J. Bex

**Affiliations:** 1 Technical University Munich, Department of Electrical and Computer Engineering, Munich, 80333, Germany; 2 Adaptive Sensory Technology, Inc., San Diego, CA 92121, USA; 3 Harvard Medical School, Schepens Eye Research Institute, Boston, MA 02114, USA; 4 Jilin University of Finance and Economics, Changchun, China; 5 The Ohio State University, Department of Psychology, Columbus, OH 43210, USA; 6 Northeastern University, Department of Psychology, Boston, MA 02115, USA

## Abstract

The contrast sensitivity function (CSF) relates the visibility of a spatial pattern to both its size and contrast, and is therefore a more comprehensive assessment of visual function than acuity, which only determines the smallest resolvable pattern size. Because of the additional dimension of contrast, estimating the CSF can be more time-consuming. Here, we compare two methods for rapid assessment of the CSF that were implemented on a tablet device. For a single-trial assessment, we asked 63 myopes and 38 emmetropes to tap the peak of a “sweep grating” on the tablet’s touch screen. For a more precise assessment, subjects performed 50 trials of the quick CSF method in a 10-AFC letter recognition task. Tests were performed with and without optical correction, and in monocular and binocular conditions; one condition was measured twice to assess repeatability. Results show that both methods are highly correlated; using both common and novel measures for test-retest repeatability, however, the quick CSF delivers more precision with testing times of under three minutes. Further analyses show how a population prior can improve convergence rate of the quick CSF, and how the multi-dimensional output of the quick CSF can provide greater precision than scalar outcome measures.

The notion that precise measurements of an outcome variable are a prerequisite to improve the underlying process goes back to at least the 19^th^ century^1^, but still holds true today. For example, *personalized medicine* currently has an unmet need for diagnostics that are precise enough to track an individual’s health state^2^. Despite a growing interest in genetic testing, behavioural tests of cognitive and sensory performance will remain important indicators of subjective experience, which ultimately determines health-related quality of life.

In the visual domain, the predominant tool for functional assessment is acuity, which measures the smallest size of a stimulus (typically, a letter) that an observer can recognize at full contrast. While acuity is well established and can be very useful e.g. for adjusting optical correction, it has at least two issues in domains where precision is paramount, such as in clinical trials. First, the variability of repeated measurements makes the method insensitive to subtle changes in vision, e.g. due to disease progression or treatment effects^3^. Second, some ophthalmic and neurologic conditions affect acuity only moderately, despite effects on visual function^4^,^5^,^6^,^7^.

A more comprehensive assay of visual performance is provided by the contrast sensitivity function (CSF). The CSF relates an observer’s ability to recognize a spatial pattern not only to its size, but also to its contrast; the behavioural CSF is also reflected in receptive field analysis of individual neurons^8^. Compared to acuity, the CSF correlates better with performance in visually guided everyday activities such as driving^9^,^10^, walking^11^, and the ability to recognize faces^12^ (for a review, see ref. 13).

In psychophysical laboratories, the CSF is typically assessed by estimating individual thresholds for a range of spatial frequencies, using one-dimensional Bayesian adaptive methods^14^. However, this precise approach is too time-consuming for regular clinical care, and the currently prevalent paper charts thus require substantial trade-offs such as coarse stimulus space resolution that might restrict variation to either spatial frequency or contrast, and simple scoring rules that can be applied manually; this has sometimes led to the introduction of idiosyncratic scoring rules, making it hard to compare results across laboratories or even individual examiners^15^,^16^. Furthermore, even for computerized testing, the upper limits of visual contrast sensitivity in healthy subjects exceed the dynamic range of consumer electronics displays, potentially leading to ceiling effects in vision tests^17^,^18^; often, cathode-ray tubes and custom electronic circuits are still being used in the laboratory to avoid such effects^19^.

However, advances in computational power and display technology have opened avenues for novel methods of visual assessment to overcome these issues. For example, the quick CSF method^20^,^21^ uses a computationally expensive algorithm and optimizes stimulus selection by computing the expected information gain over a very large set of possible stimuli and the probability distribution of possible CSFs, given the history of previous trials. Specifically, the human contrast sensitivity function can be described by four parameters - namely peak sensitivity, peak frequency, bandwidth, and low-spatial frequency truncation^22^,^23^. The quick CSF method exploits this observation and computes the probability distribution over possible CSFs, using Bayes’ theorem: 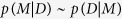, with *M* a four-dimensional tuple describing a CSF and *D* the set of trials. The range of different CSFs, each described by a different *M*, spans the entire gamut from very low to excellent vision, and initially (before the first trial, i.e. before anything is known about the subject), the algorithm assumes the same probability for each of these CSFs; alternatively, other sources of information, such as knowledge about the distribution of CSFs in a particular subject population, can be used to model a more informative initial probability distribution, or *prior*. For each trial, the algorithm then chooses that combination of stimulus frequency and contrast that maximizes expected information gain about this CSF distribution. For example, the very first trial should not be trivially easy (such as in acuity charts that start with the largest letter) because the outcome (a correct response) is so likely for almost every subject (and almost every possible CSF) that it is probably uninformative for this particular subject, and the likelihood of different CSFs. Instead, the algorithm typically chooses a medium-sized, medium-contrast stimulus for the first trial (stimulus selection is stochastic in order to prevent getting stuck in local optima), and the probability distribution over all CSFs is updated according to the subject’s response. For example, an incorrect response to such a medium-difficulty stimulus would make CSFs that represent excellent vision less likely, and thus the stimulus for the subsequent trial should not be difficult. Over the time course of the test, the quick CSF repeats these steps to home in on stimuli that are near the subject’s threshold and that provide the most information about the probability space of different CSFs. This means that a high resolution in the stimulus space (many different contrast levels and spatial frequencies) is useful because it allows the algorithm to adapt more finely to the current subject; however, not all possible stimuli need to be presented during the experiment. Finally, a sample is taken from the probability distribution of CSF models *M* and the median sensitivity over this sample can be computed for any spatial frequency based on the model parameters.

We further extended our tablet-based implementation^24^ to use bandpass-filtered Sloan letters instead of gratings. The increase in the number of possible responses (ten instead of two) reduces the guessing rate and thus increases statistical efficiency^21^. In the present paper, we evaluate the quick CSF method in a population of healthy observers, and we further investigate the effect on choosing different priors on its robustness. Implemented on an iPad^24^, our system allowed rapid testing (median testing time less than three minutes, including entering subject details) and yet precise assessment of the whole CSF.

In order to establish a baseline for how much information can be captured ultra-rapidly, in a single trial, we also collected a single-trial assessment of the peak of the CSF: using the iPad’s touch screen, observers were asked to indicate the point of highest sensitivity on a “sweep grating” picture that varies spatial frequency and contrast along the two image dimensions. We analyze the relationship between quick CSF results on the one hand and single-trial assessment of contrast sensitivity on the other hand, and also compare the test-retest variability of both assessments, using a method from information retrieval that is less prone to artefact than commonly used test-retest measures.

A necessary requirement for a precise test is that two measurements of the same true value return similar results; in other words, a precise test should be highly repeatable. However, this requirement is not sufficient, as reliable tests are not necessarily precise^25^. Commonly, test-retest variability is evaluated by the Bland-Altman Coefficient of Repeatability^26^; despite its shortcomings^27^, the intra-class correlation coefficient also is often reported.

We instead propose to assess test-retest variability based on concepts from information retrieval, namely by a measure we call Fractional Rank Precision (FRP)^28^. Intuitively speaking, we want to identify the test-retest pair of measurement for a subject, given only the test measurement for this subject and the set of retests for all subjects. If a subject’s retest score is the same as their test score again, and none of the other subjects have the same retest score, this subject’s retest is uniquely identified by the test, and we assign a precision of 1.0. Conversely, if a hypothetical retest just flipped the sign of the test, identification would be very poor, and we assign a precision of 0.0 (or for a finite number *N* of subjects, 1/*N*). Generally, each subject is assigned the fractional precision (1 − (rank − 1)/*N*) of their individual retest value when all subjects’ retest values are sorted by their similarity (e.g. Euclidean distance) to the individual test. We repeat this for each subject and FRP then is the average of all subjects’ precisions. If test and retest scores are distributed randomly, the expected FRP will be 0.5. Notably, FRP also takes into account that identical test outcomes for different subjects, e.g. due to large step sizes, reduce the ability to identify the test-retest pair. For example, in typical healthy, best-corrected cohorts, almost all subjects have one of very few different logMAR scores at or near 20/20. In the extreme, a very coarsely resolved test, such as light perception in a sighted cohort (all retest scores with the same distance to each test score), would yield an FRP of 0.5, i.e. chance performance.

As an FRP example, imagine three subjects who have test scores of 1.0, 1.5, and 1.2, and retest scores of 1.0, 1.3, and 1.4, respectively. The absolute differences of the three retest scores to the test score (1.0) of the first subject are (0.0, 0.3, 0.4) and thus the first subject’s retest is closest to its test score, i.e. has rank 1. For the test score of the second subject (1.5), the absolute differences are (0.5, 0.2, 0.1) and thus the second subject’s retest has rank 2. For the third subject (test score 1.2), the absolute differences are (0.2, 0.1, 0.2), and its retest has rank 2.5 (because first and third subjects’ tests tie for rank 2). The Fractional Rank Precision therefore is 1/3 × (1 + 2/3 + 1/2) = 0.72. This formulation has the benefit that it expresses test-retest variability in terms of inter-subject variability, without resorting to absolute values that may make it difficult to compare the repeatability of different tests with different absolute score ranges. A further benefit is the penalty for test scores that are quantized at the cost of precision^25^: a test that returns only one score may have perfect repeatability, but fails to discriminate between different subjects (and, by extension, changes due to disease progression or treatment effects).

## Results

### Single-trial assessment

**Figure 1:**
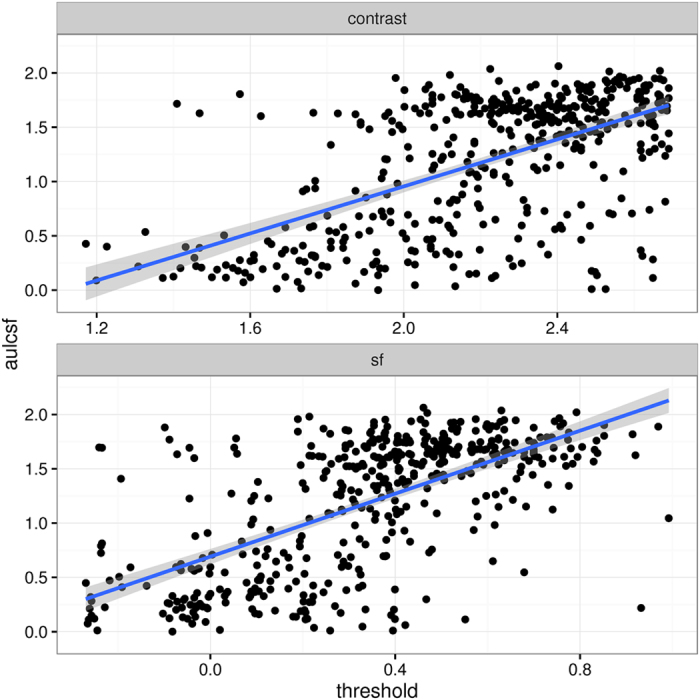
Relationship of single-trial assessment (subjects tapped the perceived peak of a sweep grating) and the outcome of the 10-AFC quick CSF algorithm after 50 trials. Top panel, peak contrast sensitivity (1/contrast) as tapped by the subject vs. AULCSF as computed by quick CSF. Bottom panel, perceived peak spatial frequency vs. AULCSF.

The two panels in Fig. 1 show the relationship of peak frequency and sensitivity as obtained by single-trial assessment on the one hand and the AULCSF of the quick CSF (after rescoring with the population prior) on the other hand. Solid lines indicate univariate linear regression and shaded regions indicate 95% confidence intervals. Both dimensions of the single-trial assessment are correlated with AULCSF (*p* ≪ 0.01, *R*^2^ = 0.34 for peak sensitivity and *R*^2^ = 0.40 for peak frequency); multivariate regression explains more of the variance (*p* ≪ 0.01, *R*^2^ = 0.54) with no statistically significant interaction between peak sensitivity and peak frequency (*p* = 0.59).

Bland-Altman Coefficients of Repeatability were 0.497 and 0.476 log10 units for peak sensitivity and frequency, respectively. Fractional Rank Precision for both features was 0.715 and 0.681, respectively; combining the two features and performing a nearest-neighbour search improved FRP to 0.769.

### Test times

**Figure 2:**
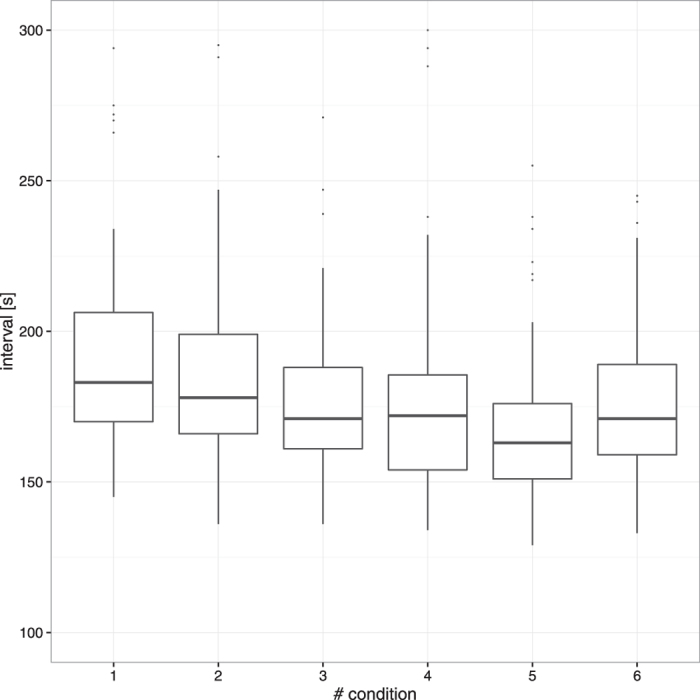
Test times plotted as a function of condition number. Median test times are about 3 minutes for entering subject details and 50 trials of quick CSF.

Test times for the quick CSF are shown in Fig. 2. These test times were computed by comparing the time stamps of condition onsets; notably, they therefore include the time between conditions where the tablet changed back and forth between subject and experimenter, data entry of subject and condition details, re-adjusting the viewing distance, and recording both single-trial assessment and quick CSF assessment (50 trials). The minimum condition interval time measured like this was 129 s; median, mean, and standard deviation were 175, 190, and 52 s, respectively, computed over all subjects. Figure 2 shows test times as a function of the temporal order of conditions; test times become shorter with practice.

### Effect of population prior

**Figure 3:**
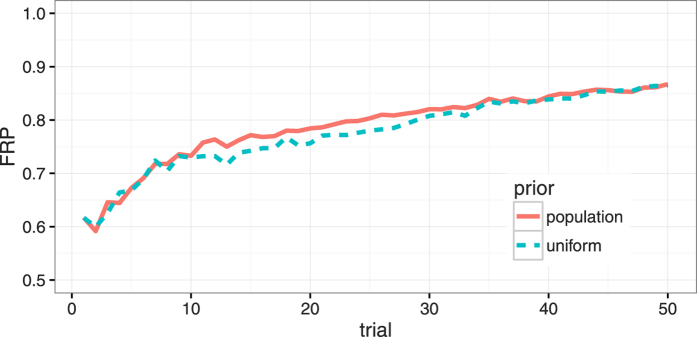
Fractional Rank Precision (FRP) plotted over the number of trials for the feature AULCSF with either uniform prior used in the actual experiment, or for data that were re-scored using a population prior. Over time, FRP increases because the quick CSF converges on the subjects’ true CSFs. This convergence is slightly faster if information about the distribution of CSFs in the population is used as a prior.

Figure 3 shows the effect of choosing a different prior over the CSF search space on Fractional Rank Precision of the summary statistic AULCSF, plotted as a function of trial number. Both the uniform and the population prior lead to very similar FRP results over the first few trials, as only very little information about the true CSF is available. For the time range between 10–30 trials, the analysis that was initialized with the population prior shows better convergence, however, as physiologically implausible parameter combinations are already suppressed. For longer test sessions, the difference between FRP for the initial priors diminishes as both approaches converge (after 50 trials, FRP of 0.867 and 0.864, respectively).

### Test-retest variability

**Figure 4:**
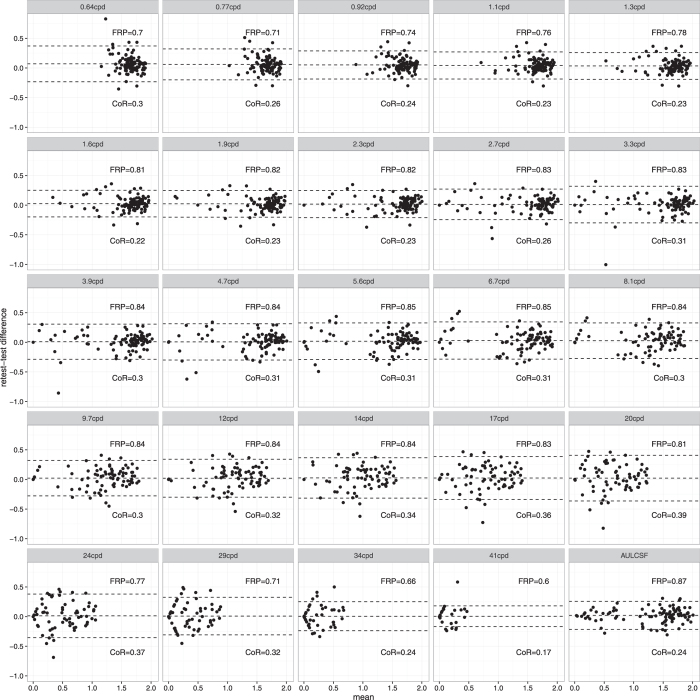
Bland-Altman plots for all 24 spatial frequencies and the summary statistic AULCSF. Text insets show Coefficients of Repeatability (1.96 times standard deviation of test-retest differences) and Fractional Rank Precision (see main text).

**Figure 5:**
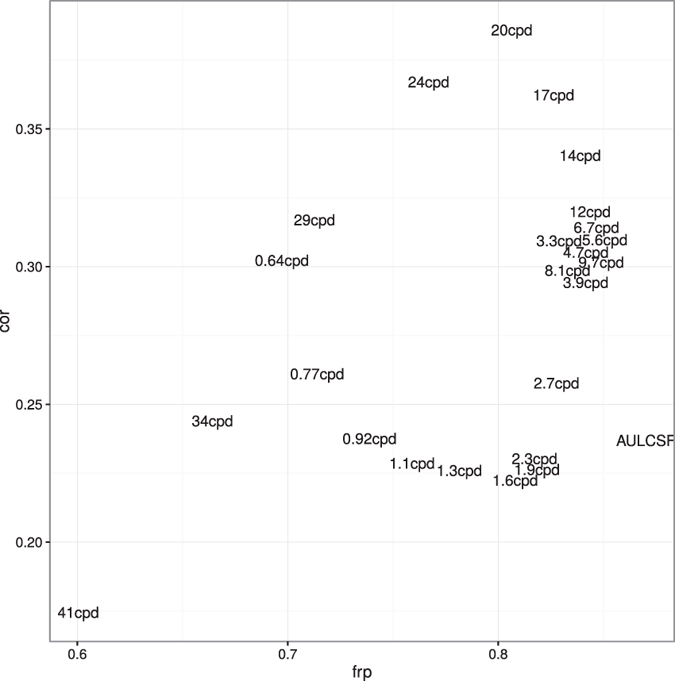
Relationship of Coefficient of Repeatability and Fractional Rank Precision for the different spatial frequencies and AULCSF. Some points have been slightly jittered for better legibility. Linear regression shows no significant relationship (*p* = 0.06, *R*^2^ = 0.15).

Figure 4 shows Bland-Altman plots and CoR and FRP values for all 24 spatial frequencies in the stimulus set and the summary statistic AULCSF. CoR and FRP have little relationship, as also demonstrated in Fig. 5 (linear regression n.s., *p* = 0.06, *R*^2^ = 0.15). A particularly strong difference between the two metrics can be seen in the bottom right of Fig. 4. Sensitivity at the highest spatial frequency 41 cpd has the lowest (best) CoR (0.17) of all features, but also the lowest (worst) FRP (0.6), whereas AULCSF has best FRP, but medium CoR (0.87 and 0.24, respectively). Along the horizontal axis, the Bland-Altman plots show that AULCSF has a much wider range than sensitivity at 41 cpd. The latter suffers from floor effects, and thus has little predictive power despite a seemingly better repeatability as measured by CoR.

**Figure 6:**
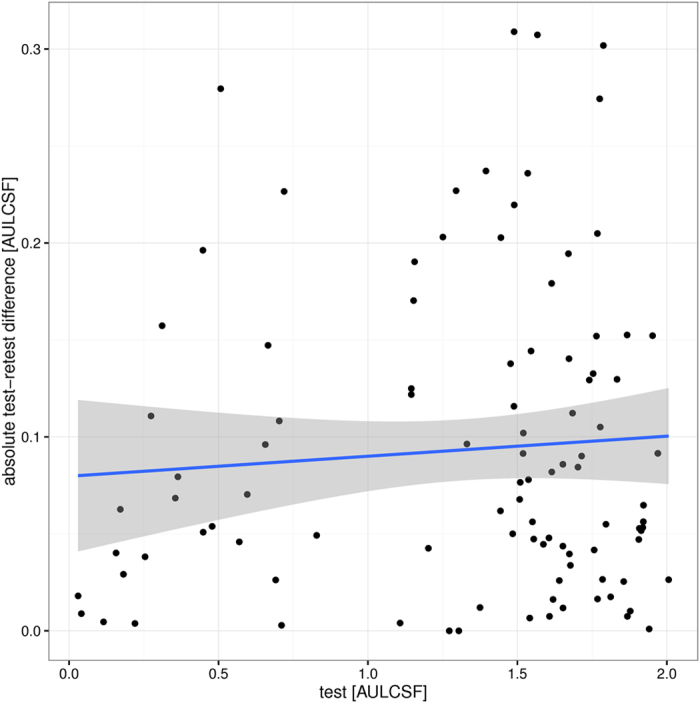
Absolute test-retest differences for the AULCSF metric as a function of visual performance (mean of test/retest scores). Statistically, subjects with poorer vision do not show worse repeatability (greater differences) than subjects with excellent vision.

Figure 6 plots the absolute test-retest difference as a function of visual performance, using the AULCSF metric. Linear regression shows no significant effect of visual function (*p* = 0.46, *R*^2^ = 0.01); in other words, subjects with poor vision exhibit similar test-retest variability as subjects with excellent vision.

### Multi-dimensional test-retest variability

**Figure 7:**
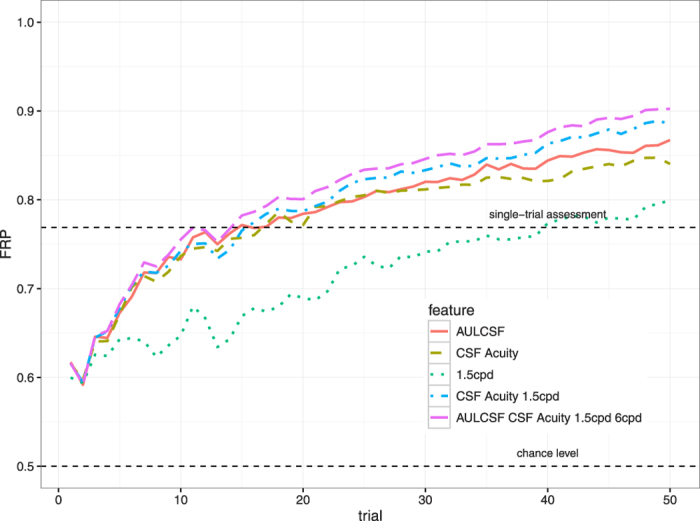
Fractional Rank Precision plotted over the time course of a test for different features and their combinations. The single-trial assessment is extremely fast, but ultimately lacks precision compared to forced-choice quick CSF measurements. Multi-dimensional features have more degrees of freedom than scalar descriptors such as AULCSF and CSF acuity and reach higher FRP levels.

Fractional Rank Precision values for different features of the quick CSF are shown in Fig. 7, with dashed lines indicating chance level (0.5) and FRP for single-trial assessment (0.769). Among scalar features, the AULCSF yields higher FRP than CSF acuity (after 50 trials, FRP of 0.867 and 0.840, respectively) as it summarizes over a large range of spatial frequencies. Bland-Altman Coefficients of Repeatability were 0.238 and 0.2 log10 units, respectively. Sensitivity at 1.5 cpd, in contrast, performed little better than single-trial assessment. Multi-dimensional features, however, identify test-retest pairs with greater precision. The combination of CSF acuity and the sensitivity near the presumed peak of the CSF (1.5 cpd) already yields a FRP of 0.886; adding the summary statistic AULCSF and an additional mid-frequency feature (6 cpd) results in the highest FRP of 0.902. Notably, FRP seems not to have reached ceiling after 50 trials for the multi-dimensional features, i.e. even more precision may be obtained by running the quick CSF method for more trials.

## Discussion

Contrast sensitivity has been recognized as a more informative outcome measure than visual acuity, the currently prevalent measure of visual function^4^,^5^,^29^,^30^,^31^,^32^. However, precise assessment of the two-dimensional CSF so far has been too time-consuming to be employed in regular clinical care; paper charts, which are limited in stimulus resolution and scoring complexity, use shortcuts that limit precision^17^,^33^,^34^,^35^.

A multitude of studies have investigated the repeatability of established contrast sensitivity charts as a proxy to precision. Absolute numbers are not necessarily comparable across studies because of different scoring rules^15^, but more importantly also because of an often observed greater test-retest variability in cohorts with lower visual performance^36^,^37^,^38^; for our test, we did not observe such heteroscedasticity (Fig. 6). Published CoR ranges for the Pelli-Robson chart include 0.13–0.21^15^, 0.14–0.41^37^ (median of individual CoRs), 0.15^39^, 0.18^40^,^41^, 0.18–0.33^42^, 0.19^38^, 0.19–0.48^36^, 0.2^34^ log10 units. Because of the coarse quantization of the Pelli-Robson chart, these numbers imply that a change in test score of at least +/−0.3 or (for low-vision patients) even 0.45 log10 is required to detect a statistically significant change. For the Mars chart, slightly lower CoRs were reported, e.g. 0.121^40^, 0.13–0.24^42^, 0.2^34^ log10 units; however, care must be taken because one of the three available Mars charts has a bias of 0.08 log10 units^34^. Repeatability studies on iPad-based tests of contrast sensitivity have resulted in CoRs of 0.19 log10 units for a letter test^38^; around 0.2 log10 units for peak sensitivity and 0.1 log10 units for peak spatial frequency based on a finger trace of a sweep grating (measured for one observer)^43^, and a range of 0.26–0.44 log10 sensitivity for individual spatial frequencies between 1.5 and 18 cpd^44^. The latter numbers are roughly comparable to the CoRs for individual frequencies observed in the present manuscript (0.17–0.39 log10 units). However, we demonstrated that the widely used Bland-Altman Coefficient of Repeatability is susceptible to scaling artefacts, and thus proposed to use Fractional Rank Precision instead to compare precision of different tests. Unlike CoR, FRP can also directly be used to compare tests that have outputs of different dimensionality. As a caveat, however, we note that FRP depends on the variability of the underlying test population, and thus FRP results cannot be compared directly across studies.

In our present study, we used two different measures of contrast sensitivity that have very short testing times, and evaluated their validity in a cohort of healthy observers with varying refractive error. The first measure required subjects to tap a tablet device only once, and therefore probably constitutes the lower limit of testing time that is practically achievable. Recently, other groups have employed a similar technique to assess contrast sensitivity^43^,^44^,^45^,^46^; these authors asked their subjects to trace out the boundary between visible and invisible pattern over a range of spatial frequencies and thus collected potentially more information than just the peak of the CSF; our single-tap measure was substantially more variable than Mulligan’s finger-tracing^43^. However, the precision of sweeping hand or finger motions is still limited and, more importantly, the subjective decision where to place the perceived boundary is likely vulnerable to criterion shifts; for these reasons, forced-choice methods are the preferred standard in psychophysical testing^47^.

Despite these fundamental problems, single-trial assessment correlated reasonably with quick CSF assessment, with an *R*^2^ > 0.5 and a test-retest precision that corresponds to about 10–15 trials of quick CSF. However, the Bland-Altman CoR was more than twice as high for peak-tapping as for the quick CSF after 50 trials, and the quick CSF in principle could be run for more trials to achieve even greater precision. Furthermore, it should be noted that the position of the sweep grating was not randomly shifted before each test session, so that subjects might have remembered (and reproduced) the location they had tapped on the screen in previous sessions^43^. Ultimately, single-trial assessment might thus serve as an ultra-rapid screening tool, but lacks the precision to track subtle changes in vision due to disease progression or treatment effects.

One potential use of such ultra-rapid screening tool might be the initialization of the quick CSF method with a subject-specific prior. We demonstrated (in Fig. 3) that the use of a population prior over the CSF parameter space already led to faster convergence than a uniform prior; furthermore, it is important to note that we here used the population prior only during a re-scoring run of the algorithm. In this case, stimulus selection was fixed and determined by the output of the quick CSF method during actual data collection, so that the rate of convergence might even be underestimated for a more informative prior. However, as discussed above, robust test-retest repeatability might come at the expense of precision to detect change, and further experiments using well-controlled changes to visual function therefore are needed to determine the optimal shape of a prior to avoid overfitting.

Kim *et al*.^48^ recently developed a Hierarchical Adaptive Design Optimization (HADO) procedure that achieves greater accuracy and efficiency in adaptive information gain, by exploiting two complementary schemes of inference (with past and future data). HADO extends the standalone quick CSF to a framework that models a higher-level structure across the population, which can be used as an informative prior for each new assessment. In turn, the parameter estimates from each individual enable the update of the higher-level structure. The judicious application of informative priors used by HADO improves the quick CSF efficiency by ≈30%. In future research, we will apply the HADO procedure as a more mathematically rigorous method to utilize population statistics to improve the efficiency and precision of quick CSF.

The quick CSF method estimates sensitivities for a wide range of spatial frequencies. The AULCSF already yields a useful summary statistic^49^,^50^,^51^,^52^,^53^,^54^, but by definition cannot exploit the full benefit of assessing the whole CSF, for example to disambiguate whether a disease might shift the CSF downwards (lower sensitivities) or left (lower peak frequency). Going beyond scalar descriptors, the combination of acuity and peak sensitivity therefore has been proposed to describe the CSF^55^. However, a priori the frequency for which sensitivity is highest cannot be known and the peak therefore must be approximated by a low-SF sensitivity. Large-scale modelling has also shown that two parameters are not enough to accurately model the CSF^23^. Such two-point measurement must assume a fixed shape of the CSF (e.g. ref. 56), which may be a reasonable first-order approximation, but likely does not hold for possible changes in optical correction, disease status, eccentricity^57^, age^22^,^58^, or illumination^44^,^59^; monocular vs. binocular testing and during development^53^,^60^.

We performed FRP analysis on higher-dimensional combinations of CSF features that might capture CSF variation more comprehensively. To this end, we used a simple nearest-neighbour approach with unit-normalized feature scales, and indeed FRP scores improved over those for scalar features such as AULCSF or CSF acuity; we note that more sophisticated Machine Learning techniques that differentially weight the individual CSF features might improve FRP scores even further. In our present analysis, best results were achieved with a combination of low-, medium-, and high-SF sensitivities (1.5 and 6 cpd and CSF acuity) and the summary statistic AULCSF; notably, this four-dimensional combination also performed better than the combination of (near-peak) sensitivity at 1.5 cpd and CSF acuity that would correspond to a two-point measurement.

In summary, we here showed that rapid assessment of the whole contrast sensitivity function on a portable device is feasible, and that the quick CSF method delivers highly precise results in testing times of less than three minutes. This efficiency can be further improved by an informed population prior or even further by the use of an ultra-rapid, single-trial prior assessment. Ultimately, the real value of a vision test is shown by its ability to detect small changes in visual function due to disease progression or treatment, and further studies are needed to evaluate the sensitivity of the quick CSF.

## Methods

### Data collection

Experimental data were collected from 101 subjects (aged 14–75 years with a mean of 22) who were recruited among students and faculty of the Institute for Psychology and Behavior, Jilin University of Finance and Economics, Changchun, Jilin Province, China. Subjects gave informed consent and the experimental design followed the principles of the Declaration of Helsinki and was approved by the local Ethics Committee (protocol IPBWH1101). Based on self-reported optical refraction, 63 subjects were myopes (mean optical correction −3.9D, s.d. 1.6D) and 38 subjects were emmetropes (no optical correction).

Subjects were tested in all applicable combinations of {right eye, left eye, both eyes} and {without correction, with correction}. To assess repeatability of our device, one randomly chosen condition was then repeated at the end of the session. While repeat measures taken over a longer time interval may be closer to clinical practice, intra-session repeatability measurement excludes subject variability over longer time scales and is thus a common method to assess the measurement itself 34,38,40,43,61,62.

Overall, 593 data sets were collected; these are publicly available at http://www.michaeldorr.de/quickcsf.

**Figure 8:**
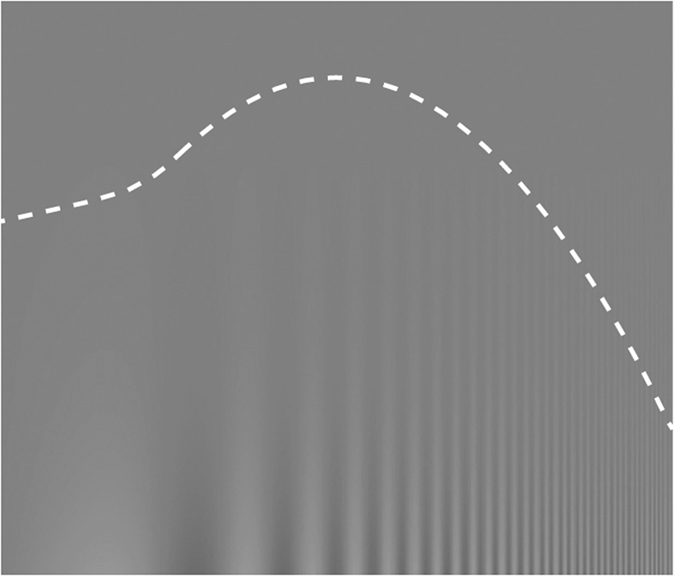
Schematic stimulus for single-trial assessment. Contrast varies along the vertical and spatial frequency along the horizontal axis. The transition from visible to invisible pattern follows the contrast sensitivity function (CSF), here exemplified by the dashed line (not shown during the experiment). Subjects were asked to touch the peak of the perceived “mountain”.

During the experiment, subjects were seated and held an iPad 4 in portrait orientation at a viewing distance of 60 cm. Mean screen luminance was set to 185 cd/*m*^2^. Prior to each tested condition, the single-trial assessment was recorded; to this end, subjects were shown a sweep grating as in Fig. 8 and asked to touch the peak of the perceived “mountain” that is created by the transition from visible to invisible contrast. The grating contrast varied vertically from 0.2% at the top of the screen to 100% at the bottom of the screen, and spatial frequency varied horizontally from 0.29 cpd at the left side of the screen to 19.2 cpd at the right.

For each of 50 trials, one of 11520 possible stimuli (10 bandpass-filtered Sloan letters, peak frequency 4.5 cycles per letter; 24 spatial frequencies from 0.64 to 41 cycles per degree; 48 contrast levels from 0.2 to 100%) was selected by the quick CSF method. The stimulus was then briefly presented (500 ms), followed by an array of letters from which subjects had to choose their response on the touch screen.

### Statistical analysis

#### Selection of prior for the quick CSF method

The quick CSF uses Bayesian inference to update its belief (i.e. the best estimate of the true CSF) after each trial. Before the first trial, however, no data have been collected and the initial belief has to be described by a *prior* probability distribution over the parameter space. Such a prior could be derived from previous knowledge, e.g. the statistics of visual function in the general population or an individual’s earlier test results. In our experiment, however, we approached data collection in the most principled way and chose a uniform distribution over the parameter space. While this makes the least assumptions, a drawback of this approach is that physiologically implausible or even impossible parameter combinations such as a very high peak frequency together with a very low bandwidth initially are equally likely as more plausible combinations, and thus convergence may be slower than theoretically possible. In order to investigate this effect after data collection had concluded, we re-scored all subjects’ responses with an additional run of the algorithm per test session. For this analysis, the posterior over the whole test set (593 data sets) was used as the prior, and we report results both for this ‘population’ prior and the original ‘uniform’ prior.

#### Test-retest variability of scalar features

From the final estimates of the CSFs, we computed threshold sensitivity for each of the 24 spatial frequencies that corresponded to our stimulus set (note that not all stimuli were necessarily shown to each observer). For a broad summary statistic, we also computed the area under the log CSF (AULCSF) in the spatial frequency range from 1.5 to 18 cpd. For these 25 features, we computed Coefficients of Repeatability (1.96 times the standard deviation of test–retest differences) and the Fractional Rank Precision metric (FRP): for each subject’s test feature *f*_subject_, we sorted all the *N* observers’ retest values *f*_*n*_ by their distance to *f*_subject_, and assigned a rank *r*(observer_*n*_) accordingly. The fractional rank for one subject then was 
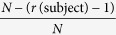
, and overall FRP was the average of fractional rank over all observers (each observer being the “subject” once):


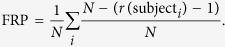


For increased robustness, FRP analysis was run in both directions and results were averaged, i.e. all retests were ranked by their distance to each test once, and all tests were ranked by their distance to each retest once.

### Test-retest variability of multi-dimensional features

By definition, the CSF is a multi-dimensional function. While the AULCSF can already provide a very useful summary statistic in many cases, higher-dimensional descriptions of the whole CSF may more precisely capture changes in the CSF and thus track an individual’s performance. We therefore performed FRP analysis on multi-dimensional feature vectors and performed a nearest-neighbour search to identify possible test-retest pairs.

In order to keep the number of possible dimensions small, we did not use all 25 features from above. Instead, we computed the following features on the CSF: i) AULCSF; ii) CSF acuity, the spatial frequency for which contrast threshold reaches 100%; iii) sensitivities for six spatial frequencies as mandated by the FDA^63^, namely 1.0, 1.5, 3, 6, 12, and 18 cpd. In order to reduce the impact of different numerical ranges, these features were standardized to zero mean and unit standard deviation, and we averaged the results of running our analysis in both directions again. For results shown in Fig. 7, we present a selection of individual features and their combinations.

## Additional Information

**How to cite this article:** Dorr, M. *et al*. Evaluation of the precision of contrast sensitivity function assessment on a tablet device. *Sci. Rep.* **7**, 46706; doi: 10.1038/srep46706 (2017).

**Publisher's note:** Springer Nature remains neutral with regard to jurisdictional claims in published maps and institutional affiliations.

## References

[b1] Thomson S. W. Electrical units of measurement. In Popular Lectures and Addresses, 73–136 (Macmillan and Co, London, 1889).

[b2] Hamburg M. A. & Collins F. S. The path to personalized medicine. New England Journal of Medicine 363, 301–304, doi: 10.1056/NEJMp1006304 (2010). 20551152

[b3] Shah N., Dakin S. C., Whitaker H. L. & Anderson R. S. Effect of scoring and termination rules on test-retest variability of a novel high-pass letter acuity chart. Investigative Ophthalmology & Visual Science 55, 1386–1392 URL http://www.iovs.org/content/55/3/1386.abstract, doi: 10.1167/iovs.13-13340 (2014). 24519424

[b4] Jindra L. F. & Zemon V. Contrast sensitivity testing: a more complete assessment of vision. J Cataract Refract Surg 15, 141–148 (1989). 2724114 10.1016/s0886-3350(89)80002-1

[b5] Woods R. L. & Wood J. M. The role of contrast sensitivity charts and contrast letter charts in clinical practice. Clinical & Experimental Optometry 78, 43–57, doi: 10.1111/j.1444-0938.1995.tb00787.x (1995).

[b6] Dosso A. A. . Risk factors associated with contrast sensitivity loss in diabetic patients. Graefes Arch Clin Exp Ophthalmol 234, 300–305 (1996). 8740250 10.1007/BF00220704

[b7] Fletcher D. & Schuchard R. Visual function in patients with choroidal neovascularization resulting from age-related macular degeneration: the importance of looking beyond visual acuity. Optometry & Vision Science 83, 178 (2006). 16534460 10.1097/01.opx.0000204510.08026.7f

[b8] Campbell F. W. & Robson J. G. Application of Fourier analysis to the visibility of gratings. Journal of Physiology 197, 551–556 (1968). 5666169 10.1113/jphysiol.1968.sp008574 PMC1351748

[b9] Freeman E. E., Muñoz B., Turano K. A. & West S. K. Measures of visual function and time to driving cessation in older adults. Optometry & Vision Science 82, 765–773 (2005). 16127343 10.1097/01.opx.0000175008.88427.05

[b10] Owsley C. & McGwin G. Vision and driving. Vision Res 50, 2348–2361 URL http://www.ncbi.nlm.nih.gov/pmc/articles/PMC2975746/, doi: 10.1016/j.visres.2010.05.021 20580907[pmid] (2010). 20580907 PMC2975746

[b11] Geruschat D. R., Turano K. A. & Stahl J. W. Traditional measures of mobility performance and retinitis pigmentosa. Optometry & Vision Science 75, 525–537 (1998). 9703042 10.1097/00006324-199807000-00022

[b12] West S. K. . How does visual impairment affect performance on tasks of everyday life? The SEE project. Archives of Ophthalmology 120, 774–780 URL + http://dx.doi.org/10.1001/archopht.120.6.774, doi: 10.1001/archopht.120.6.774 (2002). 12049583

[b13] Owsley C. Contrast sensitivity. Ophthalmol Clin North Am 16, 171–177 (2003). 12809156 10.1016/s0896-1549(03)00003-8

[b14] Watson A. B. & Pelli D. G. QUEST: A Bayesian adaptive psychometric method. Perception & Psychophysics 33, 113–120 (1983). 6844102 10.3758/bf03202828

[b15] Elliott D., Bullimore M. & Bailey I. Improving the reliability of the Pelli-Robson contrast sensitivity test. Clinical Vision Sciences 6, 471–475 (1991).

[b16] Elliott D. B. & Whitaker D. Clinical contrast sensitivity chart evaluation. Ophthalmic and Physiological Optics 12, 275–280 (1992). 1454362

[b17] Koefoed V. F., Baste V., Roumes C. & Høvding G. Contrast sensitivity measured by two different test methods in healthy, young adults with normal visual acuity. Acta Ophthalmologica 93, 154–161 URL http://dx.doi.org/10.1111/aos.12487, doi: 10.1111/aos.12487 (2015). 25056525

[b18] Wang L. . Mapping the structure of perceptual and visual–motor abilities in healthy young adults. Acta Psychologica 157, 74–84 (2015). 25747573 10.1016/j.actpsy.2015.02.005

[b19] Li X., Lu Z. L., Xu P., Jin J. & Zhou Y. Generating high gray-level resolution monochrome displays with conventional computer graphics cards and color monitors. Journal of Neuroscience Methods 130, 9–18 (2003). 14583400 10.1016/s0165-0270(03)00174-2

[b20] Lesmes L. A., Lu Z.-L., Baek J. & Albright T. D. Bayesian adaptive estimation of the contrast sensitivity function: The quick CSF method. Journal of Vision 10 URL http://www.journalofvision.org/10/3/17, doi: 10.1167/10.3.17 (2010). PMC4439013 20377294

[b21] Hou F., Lesmes L., Bex P., Dorr M. & Lu Z.-L. Using 10AFC to further improve the efficiency of the quick CSF method. Journal of Vision 15, 1–18 (2015). 10.1167/15.9.2 PMC4581618 26161631

[b22] Rohaly A. M. & Owsley C. Modeling the contrast-sensitivity functions of older adults. J Opt Soc Am A 10, 1591–1599 (1993). 8350148 10.1364/josaa.10.001591

[b23] Watson A. B. & Ahumada A. J. A standard model for foveal detection of spatial contrast. Journal of Vision 5, 717–740 URL http://journalofvision.org/5/9/6/, doi: 10:1167/5.9.6 (2005). 16356081 10.1167/5.9.6

[b24] Dorr M., Lesmes L., Lu Z.-L. & Bex P. Rapid and reliable assessment of the contrast sensitivity function on an iPad. Investigative Ophthalmology & Visual Science 54, 7266–7273 (2013). 24114545 10.1167/iovs.13-11743 PMC4589140

[b25] Bailey I. L., Bullimore M. A., Raasch T. W. & Taylor H. R. Clinical grading and the effects of scaling. Investigative Ophthalmology & Visual Science 32, 422 URL +http://dx.doi.org/ (1991). 1993595

[b26] Bland J. M. & Altman D. G. Statistical methods for assessing agreement between two methods of clinical measurement. Lancet 1, 307–310 (1986). 2868172

[b27] McAlinden C., Khadka J. & Pesudovs K. Statistical methods for conducting agreement (comparison of clinical tests) and precision (repeatability or reproducibility) studies in optometry and ophthalmology. Ophthalmic and Physiological Optics 31, 330–338 (2011). 21615445 10.1111/j.1475-1313.2011.00851.x

[b28] Dorr M. . New precision metrics for contrast sensitivity testing. 10.1109/JBHI.2017.2708745 PMC6706861 28650831

[b29] Howes S. C., Caelli T. & Mitchell P. Contrast sensitivity in diabetics with retinopathy and cataract. Australian Journal of Ophthalmology 10, 173–178 (1982). 7181757 10.1111/j.1442-9071.1982.tb00380.x

[b30] Ross J. E., Bron A. J. & Clarke D. D. Contrast sensitivity and visual disability in chronic simple glaucoma. British Journal of Ophthalmology 68, 821–827 (1984). 6498136 10.1136/bjo.68.11.821 PMC1040478

[b31] Sabour-Pickett S. . Visual performance in patients with neovascular age-related macular degeneration undergoing treatment with intravitreal ranibizumab. Journal of Ophthalmology 2013, 1–7 (2013). 10.1155/2013/268438 PMC3595676 23533703

[b32] Sebag J., Sadun A. A. & Pierce E. A. Paradigm shifts in ophthalmic diagnostics. Trans Am Ophthalmol Soc 114, WP1 URL http://www.ncbi.nlm.nih.gov/pmc/articles/PMC5141845/. 1545_6110-v114-wp1[PII] (2016). PMC5141845 28008209

[b33] Reeves B. C., Wood J. M. & Hill A. R. Vistech VCTS 6500 charts – within- and between-session reliability. Optometry & Vision Science 68, 728–37 (1991). 1745500 10.1097/00006324-199109000-00010

[b34] Dougherty B. E., Flom R. E. & Bullimore M. A. An evaluation of the Mars letter contrast sensitivity test. Optometry and Vision Science 82, 970–5 (2005). 16317373 10.1097/01.opx.0000187844.27025.ea

[b35] van Gaalen K. W., Jansonius N. M., Koopmans S. A., Terwee T. & Kooijman A. C. Relationship between contrast sensitivity and spherical aberration. Journal of Cataract & Refractive Surgery 35, 47–56 URL http://dx.doi.org/10.1016/j.jcrs.2008.09.016. doi: 10.1016/j.jcrs.2008.09.016 (2009). 19101424

[b36] Kiser A. K., Mladenovich D., Eshraghi F., Bourdeau D. & Dagnelie G. Reliability and consistency of visual acuity and contrast sensitivity measures in advanced eye disease. Optometry & Vision Science 82, 946–954 (2005). 16317369 10.1097/01.opx.0000187863.12609.7b

[b37] Bittner A. K., Jeter P. & Dagnelie G. Grating acuity and contrast tests for clinical trials of severe vision loss. Optom Vis Sci 88, 1153–1163 URL http://www.ncbi.nlm.nih.gov/pmc/articles/PMC3183246/, doi: 10.1097/OPX.0b013e3182271638 21747309[pmid] (2011). 21747309 PMC3183246

[b38] Kollbaum P. S., Jansen M. E., Kollbaum E. J. & Bullimore M. A. Validation of an iPad test of letter contrast sensitivity. Optometry and Vision Science 91, 291–6 (2014). 24413274 10.1097/OPX.0000000000000158

[b39] Elliott D. B., Sanderson K. & Conkey A. The reliability of the Pelli-Robson contrast sensitivity chart. Ophthalmic and Physiological Optics 10, 21–24 (1990). 2330208

[b40] Thayaparan K., Crossland M. D. & Rubin G. S. Clinical assessment of two new contrast sensitivity charts. British Journal of Ophthalmology 91, 749–752 (2007). 17166891 10.1136/bjo.2006.109280 PMC1955579

[b41] Bittner A. K., Ibrahim M. A., Haythornthwaite J. A., Diener-West M. & Dagnelie G. Vision test variability in retinitis pigmentosa and psychosocial factors. Optometry and vision science: official publication of the American Academy of Optometry 88, 1496 (2011). 21946786 10.1097/OPX.0b013e3182348d0b PMC3223543

[b42] Haymes S. A. . The letter contrast sensitivity test: Clinical evaluation of a new design. Investigative Ophthalmology & Visual Science 47, 2739 URL +http://dx.doi.org/10.1167/iovs.05-1419, doi: 10.1167/iovs.05-1419 (2006). 16723494

[b43] Mulligan J. B. A method for rapid measurement of contrast sensitivity on mobile touch-screens. In Human Vision and Electronic Imaging, Proc. SPIE, HVEI–104.1–HVEI–104.6 (2016).

[b44] Kingsnorth A., Drew T., Grewal B. & Wolffsohn J. S. Mobile app Aston contrast sensitivity test. Clinical and Experimental Optometry 99, 350–355 URL http://dx.doi.org/10.1111/cxo.12362, doi: 10.1111/cxo.12362 Kingsnorth15–140 (2016). 27291146

[b45] Tardif J., Watson M., Giaschi D. & Gosselin F. Measuring the contrast sensitivity function in just three clicks. Journal of Vision 16, 966 (2016).

[b46] Malmqvist L. D. & Söderberg P. G. The Uppsala Contrast Sensitivity Test (UCST): A fast strategy for clinical assessment of contrast sensitivity. In Proc. SPIE, vol. 8930, 89300H–89300H–8 (2014).

[b47] Brindley G. S. Physiology of the Retina and Visual Pathway (Williams and Wilkens, 1970).

[b48] Kim W., Pitt M., Lu Z.-L., Steyvers M. & Myung J. I. Hierarchical adaptive approach to optimal experimental design. Neural Computation 26, 2465–2492 (2014). 25149697 10.1162/NECO_a_00654 PMC4275799

[b49] Applegate R., Hilmantel G. & Howland H. Area under the log contrast sensitivity function: A concise method of following changes in visual performance. OSA Technical Digest Series 1, 98–101 (1997).

[b50] Applegate R. A., Howland H. C., Sharp R. P., Cottingham A. J. & Yee R. W. Corneal aberrations and visual performance after radial keratotomy. Journal of Refractive Surgery 14, 397 (1998). 9699163 10.3928/1081-597X-19980701-05

[b51] Oshika T., Okamoto C., Samejima T., Tokunaga T. & Miyata K. Contrast sensitivity function and ocular higher-order wavefront aberrations in normal human eyes. Ophthalmology 113, 1807–1812 URL http://dx.doi.org/10.1016/j.ophtha.2006.03.061, doi: 10.1016/j.ophtha.2006.03.061 (2006). 16876865

[b52] Yamaguchi T. . Factors affecting contrast sensitivity with the artisan phakic intraocular lens for high myopia. Journal of Refractive Surgery 25, 25–32 (2009). 19244950 10.3928/1081597X-20090101-05

[b53] Kalia A. . Development of pattern vision following early and extended blindness. Proceedings of the National Academy of Sciences 111, 2035–2039 (2014). 10.1073/pnas.1311041111 PMC3918801 24449865

[b54] Zocher M. T. . Biometry and visual function of a healthy cohort in Leipzig, Germany. BMC Ophthalmology 16, 79 URL http://dx.doi.org/10.1186/s12886-016-0232-2, doi: 10.1186/s12886-016-0232-2 (2016). 27268271 PMC4895813

[b55] Pelli D. G. & Robson J. G. Are letters better than gratings? Clinical Vision Sciences 6, 409–411 (1991).

[b56] Chung S. T. L. & Legge G. E. Comparing the shape of contrast sensitivity functions for normal and low vision. Invest Ophthalmol Vis Sci 57, 198–207 URL http://www.ncbi.nlm.nih.gov/pmc/articles/PMC4727522/, doi: 10.1167/iovs.15-18084 26795826[pmid] (2016). 26795826 PMC4727522

[b57] Rosén R., Lundström L., Venkataraman A. P., Winter S. & Unsbo P. Quick contrast sensitivity measurements in the periphery. Journal of Vision 14, 3 URL +http://dx.doi.org/10.1167/14.8.3, doi: 10.1167/14.8.3 (2014). 24993017

[b58] Owsley C. Aging and vision. Vision Res 51, 1610–1622 URL http://www.ncbi.nlm.nih.gov/pmc/articles/PMC3049199/. doi: 10.1016/j.visres.2010.10.020 20974168[pmid] (2011). 20974168 PMC3049199

[b59] Jung J. W. . Effect of the pigment-free optical zone diameter of decorative tinted soft contact lenses on visual function. British Journal of Ophthalmology 100, 633–637 URL http://bjo.bmj.com/content/100/5/633.abstract, doi: 10.1136/bjophthalmol-2015-306731 (2016). 26377415

[b60] Peterzell D. H., Werner J. S. & Kaplan P. S. Individual differences in contrast sensitivity functions: Longitudinal study of 4-, 6-and 8-month-old human infants. Vision research 35, 961–979 (1995). 7762153 10.1016/0042-6989(94)00117-5

[b61] Lee H. K. . Reproducibility of morphoscopic contrast sensitivity testing with the visual capacity analyzer. Journal of Cataract & Refractive Surgery 29, 1776–1779 URL http://dx.doi.org/10.1016/S0886-3350(03)00044-0, doi: 10.1016/S0886-3350(03)00044-0 (2003). 14522300

[b62] Wu Z. . Measurement of retinal sensitivity on tablet devices in age-related macular degeneration. Transl Vis Sci Technol 4, 13 URL http://www.ncbi.nlm.nih.gov/pmc/articles/PMC4497484/, doi: 10.1167/tvst.4.3.13 25909036[pmid] (2015). PMC4497484 26175959

[b63] Z80, A. N. S. I. C. American National Standard for Ophthalmics: Multifocal Intraocular Lenses (Optical Laboratories Association, 11096 Lee Highway, Fairfax, VA, 2007).

